# Breaking the Cycle: First‐Generation College Nursing Students Attending a Rural Community College

**DOI:** 10.1002/nop2.70878

**Published:** 2026-10-12

**Authors:** Alvaro Tellez Arriaga, Elizabeth Castillo, Garrett K. Chan, Laura M. Wagner

**Affiliations:** ^1^ Zuckerberg San Francisco General Hospital and Trauma Center San Francisco California USA; ^2^ UCSF School of Nursing San Francisco California USA; ^3^ Community Medical Centers East Main Clinic and Waterloo Clinics Stockton California USA; ^4^ HealthImpact Oakland California USA; ^5^ FirstGenRN San Francisco California USA

**Keywords:** associate degree, college, first‐generation, higher education, registered nurse, rural

## Abstract

**Aim:**

This study describes the physical, mental, social, economic, and diversity issues experienced by rural first‐generation college nursing students.

**Design and Methods:**

This cross sectional study was conducted at a rural community college situated in an agricultural area with a significant Latinx farm worker population. A total of 87 students matriculated in an associate degree RN program were invited to participate in a survey to explore a variety of issues impacting students.

**Results:**

An overwhelming majority of participants (75.6%) reported family depending on them as a source of emotional support while 47.6% also had family depending on them for financial support while attending school. Approximately 73% of participants were unsure of receiving mentoring support or did not receive mentoring support during the admissions application process. A substantial number of respondents (31.6%) had financial concerns and considered dropping out of college. First‐generation college students were significantly more likely to express an interest to work in rural settings (87.9%) following graduation (*χ* ^2^ = 9.85; df = 2; *p* = 0.007). Many first‐generation college nursing students expressed breaking down the barriers of stigma and serving as an inspiration to future family members to pursue higher education.

**Public Contribution:**

First‐generation college nursing students lack several supports traditionally viewed to help students pursue a higher education. Despite this, the participants noted they were making a difference among themselves, their family, and the rural community from which they hail.

## 1 Background and Significance

### 1.1 Decline in the Nurse Workforce

In 2021, the number of nurses under 35 in the workforce plummeted by more than 100,000 in a single year, a decline significantly higher than any seen in the previous 4 decades in the United States (Auerbach et al. 2022) although updated forecasts indicate that the profession is recovering and experiencing growth following the COVID‐19 pandemic (Auerbach et al. 2024). Globally, there are substantial shortages of nurses fueled largely by an aging retiring workforce and retention issues (Buchan and Catton 2023). Numerous studies published during the COVID‐19 epidemic have reported alarmingly high registered nurse (RN) burnout rates and moral distress (Auerbach et al. 2022; Burton et al. 2023). The National Sample Survey of Registered Nurses predicts a national deficit of 78,610 full‐time equivalent (FTE) RNs in 2025 and 63,720 FTE RNs by 2030 (HRSA 2023).

Rural areas continue to be a challenge for the nursing profession, and the number of nurses required in each state varies considerably. The 10 states with the greatest anticipated labor deficits in 2035 are projected to be Washington (26%), Georgia (21%), California (18%), Oregon (16%), Michigan (15%), Idaho (15%), Louisiana (13%), North Carolina (13%), New Jersey (12%), and South Carolina (11%) (HRSA 2023). Although the nursing shortage is nationwide, the shortage is greatest in rural areas (HRSA 2023).

### 1.2 Health Care Disparities in Rural America

Approximately 46 million Americans reside in rural areas (CDC 2020; Health Resources and Service Administration 2021), and disparities in access to health care in rural settings are a significant concern for the nation as a whole (Basu et al. 2019). In comparison with urban residents, rural residents are more likely to die prematurely from the five primary causes of death: cardiovascular disease, cancer, unintentional injury, chronic lower respiratory disease, and stroke. Rural residents are also more likely to be adversely affected by barriers to health care such as long distance to health care facilities and inadequate transportation to medical care, privacy and social stigmatization, low health insurance rates, low levels of health literacy, and health care worker shortages (NIHCM 2023). Disparities also include access to health care, health practices, and even availability to healthy foods (CDC 2020). A lack of access leads to poorer health outcomes, higher rates of chronic illnesses, and less monitoring of care (Mainous et al. 2004). In addition, rural populations had significantly higher rates of opioid abuse and deaths from 2012 to 2019, with a 45% higher mortality rate due to opioid overdose in rural settings compared to urban areas (Hedegaard and Spencer 2021). The COVID‐19 pandemic also heavily impacted rural areas' public health and health care with worsening disparities—such as lower vaccination rates, higher mortality and hospitalization rates (Mueller et al. 2021).

### 1.3 Farmworkers' Poor Health Outcomes

Farmworkers in rural health settings are at particular risk for poor health outcomes; the average life expectancy of a farm worker in the United States is 49 years old (Center for Farmworker Families 2023). Chronic disease risk factors identified in the California Agricultural Workers Health Survey include overweight–obesity (81% of farmworkers) and elevated rates of high cholesterol, high blood pressure (particularly in the 20–44 year‐old age group) and diabetes all of which exceed those of the general U.S. population. Also, more than a quarter of farmworkers report having had occupational injury (Villarejo et al. 2010). Exposures to pesticides and other occupational hazards, oral health problems such as caries and periodontal disease, infectious diseases such as tuberculosis and sexually transmitted infections, including HIV disease, and mental health problems such as depression and anxiety all disproportionately impact migrant and seasonal workers. These chronic diseases accelerate the aging process, and their disparate prevalence's in the rural community's older adults underscore the need for rural communities to be better prepared to care for members of this older age group. Eldercare workforce issues in rural areas specifically include not only the lack of an adequate workforce but also a lack of proven strategies for effectively recruiting a health care worker earlier in their career who would be motivated to care for older adults (Arcury and Quandt 2020).

### 1.4 Impact of Nurse Workforce Shortages on Rural Nursing Homes

Rural nursing homes have been especially impacted by pandemic‐related workforce shortages. As the pandemic progressed, the shortages of personnel—particularly direct care workers—escalated significantly in rural areas (Yang et al. 2021). These findings highlight the importance of establishing measures to prevent rural nursing homes from having personnel shortages in the aftermath of long‐term natural disasters such as the COVID‐19 pandemic. Nursing homes have long struggled with staffing shortages, but the COVID‐19 pandemic has exacerbated the problem. Moreover, staffing shortages have been reported in both rural and urban nursing homes, but according to the Kaiser Family Foundation, rural nursing homes have experienced more severe staffing shortages than urban nursing homes during the pandemic (Yang et al. 2021).

### 1.5 Increasing the Rural Nursing Workforce

There are multiple types of professional nurses who work in rural settings; these include licensed vocational nurses/licensed practical nurses and RNs. Each nurse has a different level of education, from an associate degree to a bachelor's degree and even a master's degree or doctoral degree. Associate degree nursing programs may be more feasible for a first‐generation college student due to affordability and shorter time to completion compared to a bachelor's degree. In order to address the ongoing staffing shortages in health care settings, increasing the supply of nurses in rural areas is critical. The shortage of health care providers in rural areas is a multifaceted problem that requires a multifaceted solution. Potential solutions may include increasing funding for rural health care facilities, incentivizing health care providers to work in rural areas, programs that train and retain within the local community, and expanding educational opportunities to first‐generation college undergraduate and graduate nursing students. However, implementing these solutions and resolving the rural workforce shortage problem will require concerted efforts from policymakers, health care providers, and community members alike.

### 1.6 Improving Rural Nurses' Levels of Educational Attainment

Over time, the educational attainment of people living in rural areas has increased, but has not kept pace with that of urban areas, especially in terms of attainment of college and graduate degrees. To fulfil increasing health care demands, especially in rural areas, rural areas must deploy greater numbers of nurses for practice in a variety of health care settings in order to ensure that rural areas have an adequate supply of nurses who can provide care with the necessary knowledge, skills, and competencies (Alshahrani 2022). With modest growth in the number of students finishing RN pre‐licensure programs each year over the past few years, interest in nursing as a profession and enrollment of new students in RN programs remain high (Yates et al. 2023).

As a result of these deficiencies in health care access and quality, one solution to the workforce health care crisis in rural areas is to recruit nursing students who are the first in their families to attain a college degree given that only 21.1% of rural Americans earned a Bachelor's degree or higher, compared to 35.5% of Americans living in non‐rural areas (Postsecondary National Policy Institute 2023). As a group, first‐generation college graduates have multiple intersectionalities and identities based on their gender, race, ethnicity, income, and geographic location that may differ from students whose parents are college graduates. Furthermore, first‐generation college healthcare providers may have a unique perspective on the experiences of patients from similar backgrounds, resulting in better patient‐provider relationships and health outcomes (Foltz et al. 2025). Providing patients with access to healthcare providers who understand their cultural and linguistic needs can aid in the reduction of healthcare disparities and the improvement of overall health outcomes (Jetty et al. 2022; Wagner et al. 2020).

When it comes to recruitment, mentoring, and retention, nurses who are the first in their families to attend and graduate college may also face unique obstacles. These obstacles may include a lack of knowledge about the higher education system, limited access to resources and support, and feelings of isolation or imposter syndrome in academic and professional settings—they are essential to improving our nation's health care. In contrast to a Bachelor of Nursing Science (BSN), which traditionally takes place at a 4‐year university, an Associate's Degree in Nursing (ADN) is an alternative pathway to becoming a registered nurse. Most ADN programs are 2 years in length and located within community colleges. ADN programs may be more suitable for first‐generation college students as they are more affordable, provide more flexible arrangements to accommodate familial (e.g., caregiving) responsibilities, and are affordable (Fontenot and Mastorovich 2024).

Little is known about how to support first‐generation college nursing students, their challenges, and their opportunities for successful academic program towards degree attainment. Therefore, in order to better assist this rising RN workforce, this study describes a sample of first‐generation college nursing students who will be able to fill the persisting deficiencies in rural patient care. Specifically, the study describes the physical, mental, social, economic, and diversity issues experienced by rural first‐generation college nursing students.

## 2 Methods

### 2.1 Setting

This study was conducted at a California (U.S.) rural community college situated in an area with a significant Latinx population at 79.6%. Only 10% or less of the area's residents have a bachelor's degree or higher compared to 23% nationally (United States Census Bureau 2022). The college's location in a rural, agricultural region attracts students from the neighbouring agricultural regions. The area is the nation's leading region for the production of fresh market vegetables; vegetables and fruits are cultivated on more than 0.275 million acres of the area's land. The college also sits in a region that is designated by the Health Resources and Service Administration to be both a Medically Underserved Area and a Health Professional Shortage Area (HPSA) (Health Resources and Services Administration 2023). The term Medically Underserved Area refers to a geographic location whose population does not have adequate access to primary care services. These populations may be confronted by financial, cultural, or linguistic hurdles to health treatment. Health Professional Shortage Areas are areas that lack an adequate number of providers (e.g., primary, dental, and/or mental health care professionals) for the entire population (Health Resources and Services Administration 2023). The Rural–Urban Commuting Area Code classification system was further used to define the clinical settings as rural (RUCA = 4) (USDA ERS 2023).

### 2.2 Sample

In April 2022, a total of 87 students from the first‐ and second‐year cohorts of a rural associate degree RN program part of a community college were invited to participate in the study. Students reside in the greater Central Coast of California (Thorman et al. 2023). Institutional Review Board (IRB) approval was acquired from the researchers' health sciences university (IRB Number: 17‐2279; last updated December 11, 2024). The university established a formal agreement with the community college. The director of the RN program was responsible for communicating and disseminating information to students (e.g., recruitment flyer), however, there was an ‘arms‐length’ relationship whereby the RN program was not involved in the recruitment nor retention of students participating in the project in order to minimize potential bias or power issues.

### 2.3 Survey

A cross‐sectional survey was developed through multiple stages of pilot testing and development and using standard questions from already validated national surveys (Health Impact 2021). The data centered on demographic information, such as age, birthplace, and race. The term ‘first‐generation college student’ was operationalized using the Higher Education Act (1965) definition:

[a] *n* individual both of whose parents did not complete a baccalaureate degree; or…,

[i] *n* the case of any individual who regularly resided with and received support from only one parent, an individual whose only such parent did not complete a baccalaureate degree (p. 3–4) (United States Congress House 1967).

To obtain pre‐administration feedback on the survey, three first‐generation college nursing students not associated with the institution piloted the survey and provided feedback about wording, flow, and timing. Following completion of the pilot, the program administrator sent several emails and three reminders to the 87 students. The email included an attached letter that described the survey; to enable students to access the survey, the body of the email also included both a hyperlink and a quick response (QR) code. The survey was deployed through Survey Monkey, and student responses were anonymous and confidential. Participants were exempt from providing consent. Their return of the anonymous survey indicated their consent. Students received a $25 Amazon gift card for completing the survey.

The questionnaire's final element consisted of an open‐ended question designed to elicit qualitatively assessed responses from the student nurse participants. The question posed was ‘What does it mean (i.e., to elicit a wide range of responses) to be a first‐generation college nursing student?’ Given the limited research conducted on the experiences of first‐generation college nursing students, participants were given the opportunity to elaborate on their survey replies regarding their perceptions and roles as first‐generation college student nurses.

### 2.4 Data Analysis

Data analysis included descriptive and inferential statistics (e.g., chi‐square, *t*‐tests) aimed at describing the sample of first‐generation student applicants as well as comparing first‐generation students to those whose parents received at least a university degree. The survey data were analysed using IBM SPSS Statistics version 29 (Berger and Guidroz 2010; Kelly 2009). The intersectionality framework was used to guide this study in how the hierarchies of race, gender, and class inform the nursing profession regarding issues of social injustice and inequities (Berger and Guidroz 2010; Kelly 2009).

Qualitative content analysis of the open‐ended questionnaire item was guided by methods described by Berg (Health Resources and Services Administration 2023) and by Graneheim and Lundman (USDA ERS 2023). A research assistant transcribed and uploaded the open text responses. An inductive approach to data analysis was completed whereby two research analysts independently coded the data using line‐by‐line open coding in which phrases and words were assigned codes that were meant to capture the meaning of what was being expressed (Stage 1). Stage 2 of the data analysis involved systematically collapsing and placing codes from all participant responses into their corresponding categories and related subcategories by subsuming the codes under higher order themes. To strengthen methodological rigour, we implemented a number of strategies including interrater reliability checks on codes and independent coding by two analysts. When there were discrepancies between the two researchers, a third researcher was included. Following the coding, themes were captured.

## 3 Results

A total of 82 surveys were returned indicating a 91% response rate. Approximately 70% of participants were first‐generation college students, with the remainder reporting one or both parents having at least a Bachelor's degree or higher. The majority of participants (81.7%) were female, and the races of the participants were reported as White/Caucasian (48.78%), Asian (14.6%), Multiracial (12.2%), Black (4.9%), American Indian/Alaskan Native (1.2%), and Native Hawaiian/Pacific Islander (1.2%). Ethnicity of the participants was reported as Hispanic/Latino/Latinx (50%). Only 1.2% of applicants were either active duty or veterans of the military (Table 1). One‐quarter of the respondents were fluent in Spanish with an additional 12 respondents fluent in another language (e.g., Tagalog, Cebuano). The mean age of students was 31.54 years with no significant difference between first‐generation college and those who were not [*t* = (−)0.27; *p* = 0.77].

**TABLE 1:** Demographics of students (*n* = 82).

Variables	Frequency	Percent
Age		
Gender		
Male	14	17.07
Female	67	81.71
Non‐binary	1	1.22
Race		
American Indian/Alaskan Native	1	1.22
Asian (all categories)	12	14.63
African American/Black	4	4.88
Native Hawaiian/Pacific Islander	1	1.22
White/Caucasian and Middle Eastern	40	48.78
Multi racial/ethnic	10	12.20
Ethnicity		
Hispanic or Latino	41	50.00
Non‐Hispanic or Latino	41	50.00
Primary language		
English	49	59.76
Spanish	21	25.61
Tagalog	4	4.88
Other	8	7.76
Military		
No	81	98.78
Yes	1	1.22
Semester		
1st semester	30	24.39
2nd semester	34	41.46
3rd semester	4	4.88
4th semester	24	29.27
Plan to work in an under‐resourced community after program		
No	1	1.22
Yes	33	40.24
Unsure	48	58.54
My family depends on me as a source of financial support		
Strongly agree	16	19.51
Agree	23	28.05
Neither agree nor disagree	15	18.29
Disagree	15	18.29
Strongly disagree	13	15.85
Received mentoring support during admission application process		
No	49	59.76
Yes	21	25.61
Unsure	11	11
Other	1	1
Mother's highest education		
Do not know	5	6.10
Grade school	21	25.61
Some high school (did not complete)	13	15.85
High school diploma or GED	19	23.17
Some college (did not complete)	13	15.85
Associate's Degree	9	10.98
Bachelor's Degree	15	18.29
Some graduate school (did not complete)	1	1.22
Master's Degree	5	6.10
Doctoral Degree	0	0.00
Father's highest education		
Do not know	6	7.32
Grade school	14	17.07
Some high school (did not complete)	9	10.98
High school diploma or GED	20	24.39
Some college (did not complete)	11	13.41
Associate's Degree	7	8.54
Bachelor's Degree	11	13.41
Some graduate school (did not complete)	1	1.22
Master's Degree	3	3.66
Doctoral Degree	2	2.44
RN prelicensure program type		
Associate degree in Nursing (ADN)	66	80.49
Associate degree in Nursing (LVN to ADN)	16	19.51
Family's current economic status		
Long‐term poverty	6	7.79
Working class	22	28.57
Lower middle class	22	28.57
Middle class	19	24.68
Upper middle class	3	3.90
Wealthy	0	0.00
Unsure	5	6.49
Academic enrollment status		
Full‐time	70	90.91
Part‐time	7	9.09

Of the students who completed the survey, 24.4% were in their first semester, 41.4% second semester, 4.9% third semester, and 29.3% in the fourth and final semester of their nursing program. Significant factors influencing participants' decision to attend college included both parent/guardian encouragement (53.7%) as well as self‐motivation (89.0%).

An overwhelming majority of participants (75.6%) reported family depending on them as a source of emotional support while 47.6% also had family depending on them for financial support while attending school. Common barriers reported by nursing students in the study included the application process, tuition costs, accessing school counsellors, lack of information, and school distance. Approximately 73% of participants were unsure of receiving mentoring support or did not receive mentoring support during the admissions application process, while only 25.6% did. During the first weeks of nursing school, 15.9% of the participants felt closely connected with faculty members, while 46.3% felt out of place; conversely, 62.2% reported feeling connected with one or more of their peers. Although in nursing school, 52.4% of respondents reported an increase in feeling closely connected with faculty members, 12.2% remained feeling out of place, and 78.1% reported feeling connected with one or more of their peers. Yet, when comparing first‐generation to non‐first‐generation students, none of these barriers differed between the groups (*p* > 0.05) with the exception of one dimension. First‐generation college students were more likely (94.4%) to be questioned about their future potential by high school guidance counsellors than non‐first‐generation college students (5.6%) (*χ* ^2^ = 6.43; df = 1; *p* = 0.01) (Table 2).

**TABLE 2:** First‐generation college applicant comparisons.

		Not first‐generation college *n* (%)	First‐generation college *n* (%)	*p*	*χ* ^2^ (df)
Ethnicity Hispanic/Latino		5 (12.5)	35 (87.5)	< 0.001	11.12 (1)
Non‐Hispanic/Latino		19 (46.3)	22 (53.7)		
Work in under resourced setting					
Unsure		19 (40.4)	28 (59.6)	0.007	9.85 (2)
Yes		4 (12.1)	29 (87.9)		
Grew up rural/low‐income					
No		21 (37.5)	35 (62.5)	0.020	5.39 (1)
Yes		3 (12.0)	22 (88.0)		
Potential questioned by high school counsellor					
No		23 (36.5)	40 (63.5)	0.011	6.43 (1)
Yes		1 (5.6)	17 (94.4)		

Common funding sources reported by respondents included federal or state loans (63.6%), grants (42.9%), scholarships (40.3%), parents' savings or income (18.2%), savings (71.4%), personal credit cards (44.2%), and income from work while in nursing school (57.1%). First‐generation and non‐first‐generation students were receiving similar sources of funding across all of these (*p* > 0.05).

Respondents reported working full‐time (10.4%), part‐time (29.9%), and on‐call or per diem (23.4%), while attending nursing school. A substantial number of respondents (31.6%) had financial concerns and considered dropping out of college. Over 50% (*n* = 42) of students had zero college loans with the mean amount of loans equal to $9283. Interestingly, non‐ first‐generation college students were significantly more likely to carry greater mean student loan debt ($21,404) compared to students who were first‐generation college ($4192) (*t* = 1.40; df = 69; *p* = 0.007).

Participants reported having trouble accessing health care services (36.8%), study space that supports learning (36.8%), lacking educational materials and supplies (29.0%), transportation (23.7%), and childcare (22.2%) while in nursing school. Experiences of bias or discrimination were reported by respondents regarding race, ethnicity, and national origin about 7% of the time. A total of 87.4% reported being hard workers and 90.8% reported achieving goals that took years of work. Average commute times to school (25 min) and clinical (30 min) did not significantly differ between first‐generation and non‐first‐generation college students (*t*‐tests both *p* > 0.05).

A significantly greater number of first‐generation college students grew up in a rural or designated low‐income area (88%) compared to those who were not first‐generation (12%) (chi^2^ = 5.90, df = 1, *p* = 0.02). A total of 40.2% of respondents planned to work in under‐resourced communities after finishing their schooling, with first‐generation college students significantly more likely to express an affirmative interest in working in this setting (87.9%) compared to non‐first‐generation college students (12.1%) (*χ* ^2^ = 9.85; df = 2; *p* = 0.007) (Table 2).

The respondents overwhelmingly expressed an interest in continuing their career after completing the associate degree RN program, with many students already matriculated in a RN‐BSN bridge program. Over half of the students indicated interest in clinical care such as case management, leadership, education, or advanced practice positions such as those of NPs. In 1–3 years finishing the associates in program, 89.2% of participants showed interest in completing their BSN while 59.1% planned on master's level education in nursing. 31.5% showed interest in a doctoral‐level education, and 15.4% of participants showed interest in a PhD education.

As noted in Table 3, five themes were identified using the open‐ended survey question regarding the significance of being a first‐generation college student. Themes identified included: (1) breaking the cycle (as the first‐generation college student going into higher education), (2) financial challenges, (3) empowerment and pride, (4) pressure and uncertainty, as well as (5) gratitude and motivation.

**TABLE 3:** Student responses to ‘What is a first‐generation college student?’.

Breaking the cycle	‘Being a first‐generation college student is empowering and scary. Empowering because I am able to break the chain and scary because there are many unknowns about the process’ ‘To me it means to be able to get my degree despite the obstacles that are/were presented …. Also being able to pursue higher education and be able to break the cycle of not being or able to attain a college degree and be an inspiration to other members of my family’ ‘To me being a first‐generation college student mean I have to break barriers and stigma surrounding first‐generation college students’ ‘As the oldest of my siblings, I feel that it is an opportunity to set an example and be a role model’
Financial challenges	‘First to go to get a degree and increase my opportunities in finding a job’ ‘It has been difficult. My parents are hard workers and put their family first instead of furthering their education. They moved to America for a better future. As a first‐generation college student, it is difficult through the expenses I pay for college and previous loans I had to take out so I can continue in a higher education’ ‘To graduate with an associate degree, be able to help my family, work and have opportunities that my parents never had’ ‘Financial challenges and lack of support from family who may not understand the demands of college’
Empowerment and pride	‘Being “First‐generation College” makes me feel so proud. It is a lot responsibility too because you have to push through, complete that to be able to set the role model to inspire younger people in the family’ ‘I am able to pursue things that interest me & make a career rooted in my passions. My family did not have the same opportunities that I have’ ‘It's actually an honour and I hope to motivate my son, nieces, and nephews to continue their education’ ‘Pride in knowing that I am representing my family and will be able to provide for them as they age’
Pressure and uncertainty	‘A lot of pressure is on you to succeed, even though you don't have the luxuries as other students’ ‘It means I'm the first one to receive a college education in my family. Having the support of your family but in a way still being a little lost because you don't have that guidance since this is new to both you and your family’ ‘First‐Gen means that you are the first in your family to attend a higher education institution, and venture into an unknown world of opportunity. It also means that you will be learning and experiencing a lot of new things on your own’ ‘There is a lot of expectations from me after attending college’
Gratitude and motivation	‘Being 1st generation means a lot to me because I now know that if I put my best effort I can get to my goals in life’ ‘It feels great to be first‐generation college. My parents are very proud. I've gained the admiration of those who I've most admired, my parents. Accomplishing a college degree means the whole world to me’ ‘It is an honour and a privilege after all the sacrifices my parents have made for me’ ‘It means that I have the opportunity to create a life for myself that I want’

In terms of *breaking the cycle* into higher education, many first‐generation students expressed breaking down the barriers of stigma, generational curses, and serving as inspiration to future family members to pursue higher education. Additionally, the first‐generation college students helped pave the path of a previously unknown journey of higher education. One student shared, ‘Being a first‐generation college student is empowering and scary. Empowering because I am able to break the chain and scary because there are many unknowns about the process.’

Financial struggles endorsed by student participants include having to work while completing coursework, the hidden costs of higher education, and limited familial support due to limited understanding of college demands. They also recognized the importance of higher education and many families provided direct support to the student ahead of their own needs. ‘It has been difficult. My parents are hard workers and put their family first instead of furthering their education. They moved to America for a better future. As a first‐generation college student, it is difficult through the expenses I pay for college and previous loans I had to take out so I can continue in a higher education’. Understanding the role of the financial aid office and resources available such as basic needs (e.g., food pantry) to provide support during their education was also an unknown experience.

Regarding empowerment and pride, students viewed being the first in their family to attend college as an honour and a duty to inspire younger generations to pursue their passions and serve as living examples that higher education is attainable for loved ones. ‘It's actually an honour and I hope to motivate my son, nieces, and nephews to continue their education’.

First‐generation college students also expressed they faced a great deal of pressure to succeed because they are the first in their family to attend college and will be learning and experiencing new things on their own. ‘It means I'm the first one to receive a college education in my family. Having the support of your family but in a way still being a little lost because you don't have that guidance since this is new to both you and your family’.

Being a first‐generation college student means a lot to the respondents since they reported it gives them the opportunity to achieve their goals and make a life for themselves. ‘It feels great to be first‐generation to college. My parents are very proud. I've gained the admiration of those who I've most admired, my parents. Accomplishing a college degree means the whole world to me.’

## 4 Discussion

Supporting first‐generation college nursing students in bridging the gaps for rural health care is crucial. Consequently, we must have a deeper understanding of the experiences of first‐generation college nurses. This study describes the physical, mental, social, economic, and diversity issues experienced by rural first‐generation college nursing students. Specifically, we found that the participants were first‐generation college, ethnically diverse, and resided in underserved communities lacking several supports traditionally viewed to help students pursue a higher education. Despite this, the participants were highly motivated to continue their initial RN training beyond community college and noted they were making a difference among themselves, their family, and the rural communities from which they hail.

Comparing these data to other published research on first‐generation college nurses, 3.6% of applicants identified as active duty or veteran service members (Wagner et al. 2020), whereas this study found less than half of that number at 1.2%. Compared to previous research where first‐generation college students were more likely to be older applicants, this study shows no significant difference in age between first‐generation and non‐first‐generation college students (Wagner et al. 2020). Further, previous research where 30% of students identified as first‐generation college, this study identified 70% of students identify as first‐generation college (Wagner et al. 2020). Similar demographics were seen in the student population such as the majority of students being female and of white/Caucasian race as well as those who identify as multiracial, Asian and Black. Compared to recent data, 50% of the students in this study identified as Hispanic.

A total of 80.2% of students were found to have federal loans, compared to 48.8% of students who did not, yet these differences were not significant when comparing first‐generation to non‐first‐generation college students. The significantly overall lower student loan burden for first‐generation college students was not surprising, yet there could be two reasons for this finding. First, first‐generation college students may be more likely to have greater financial aid packages thus reducing their student loan burden. Although this study did not find differences in the types of student aid (e.g., loans vs. grants) between first‐generation college and those whose parents were educated, we did not collect data on the amounts of this aid other than their student loan debt. The finding differs from other research that suggests first‐generation college students owe more in student loans compared to non‐first‐generation college students (Fry 2021). Given the increased publicity about the high student loan burden by Black and Hispanic students, further research should examine whether these student groups are less likely to take out more student loans and work more during their education (Department of Financial Protection and Innovation 2023). One limitation of this study is we did not explore the proportions of student income, living expenses, and tuition to help answer this question.

When compared to the California newly licensed RN employment survey research report from 2020, our study reveals a similar distribution of gender, students taking a leave of absence, concerns about repaying student loans or educational debt, financial concerns leading to the consideration of dropping out, and food insecurity (Health Impact 2021). In contrast, our study revealed that the number of Latino/Hispanic and African American and native Hawaiian/Pacific islander students was nearly four times that of white students. In addition, our study revealed 18.5% of students were concerned about losing their residence, compared to 11.5% who were not concerned. Our study also identified students with unstable housing; 3.7% of students compared to 1.9% did not have a stable residence.

One of the strengths of the study was that a majority of the participants were nursing students of colour. Culturally‐, socially‐, and racially‐concordant care results in significantly better health outcomes and patient satisfaction when healthcare providers ‘mirror’ the patients they serve (Blanchard et al. 2007). California has the most diverse older population in the nation (U.S. Census Bureau 2017). Latinos are California's largest ethnic group at 15 million. With the increasingly aging and diverse patient population, there is an urgent need to examine specific challenges faced by the nursing workforce to meet the demands of the future, especially since only 10% of RNs are Latinx (National Council State Boards of Nursing 2020). Given substantial racial and ethnic disparities in healthcare and the specific challenges in caring for those in rural settings, one way to bridge this gap is by increasing the number of first‐generation college nurses to improve health outcomes in rural areas (Smedley et al. 2003).

## 5 Limitations

A few limitations were identified in this study. The study was a cross‐sectional study conducted at a single rural site and focused on nursing students at a community college, which may limit its generalizability. In addition, this may further limit generalizability, as it may not reflect first‐generation college students at the Bachelor's of Science or Master's level. First‐generation college students in other regions of the country may be of a different race or ethnicity than those in this region, and they may face a variety of unique obstacles within their own region. Despite these constraints, the farmworker communities in this rural region of California were represented by a highly diverse student body.

## 6 Implications

Rural first‐generation college nursing students face unique obstacles in pursuing their educational and professional goals. The first in their families to attend college, these students may lack the support and resources available to other students. In addition, rural areas may have limited access to healthcare facilities and educational institutions, making it difficult for students to locate nursing programs that meet their needs. These students face obstacles such as limited resources, a dearth of role models, financial hardships, a great distance from educational institutions, and limited employment opportunities. Despite these obstacles, there are strategies rural first‐generation college nursing students can employ to overcome obstacles and achieve their objectives. These include seeking out mentors and support networks, using online resources and distance learning programs, and pursuing opportunities for scholarships and financial aid (UC San Francisco, n.d.).

## 7 Conclusion

Rural nursing health can play a critical role in improving health outcomes of people living in rural areas, especially farmworker communities. Targeted solutions such as building capacity of the nursing workforce within can assist stakeholders in developing targeted solutions. Opportunities to expand the workforce through recruitment and retention of first‐generation college nurses are one solution to tackling nursing workforce shortages in underserved rural areas.

## Funding

Health Resources and Services Administration Grant # 1 D19HP42696‐01‐00.

## Disclosure

The authors have nothing to report.

## Conflicts of Interest

The authors declare no conflicts of interest.

## Data Availability

The data that support the findings of this study are available on request from the corresponding author. The data are not publicly available due to privacy or ethical restrictions.
